# Influence of the Environmental Factors on the Accumulation of the Bioactive Ingredients in Chinese Rhubarb Products

**DOI:** 10.1371/journal.pone.0154649

**Published:** 2016-05-03

**Authors:** Guangxi Ren, Li Li, Huijuan Hu, Yanpeng Li, Chunsheng Liu, Shengli Wei

**Affiliations:** 1 School of Chinese Materia Medica, Beijing University of Chinese Medicine, Beijing, P. R. China; 2 Beijing Hospital of Traditional Chinese Medicine, Capital Medical University Beijing, P. R. China; 3 Beijing Institute of Chinese Medicine, No. 13 Shuiche Hutong, Xinjiekou, Xicheng District, Beijing, P. R. China; 4 Engineering Research Center of Good Agricultural Practice for Chinese Crude Drugs, Ministry of Education, Beijing, P. R. China; Chinese Academy of Medical Sciences, Peking Union Medical College, CHINA

## Abstract

To provide a basis for controlling the quality of rhubarb under artificial cultivation, the present work was designed to evaluate the contents of 14 active pharmaceutical ingredients (API) of rhubarb in major rhubarb production areas in China and analyze the correlations between the contents of API and such factors as species, geographic distribution and soil. The levels of fourteen API in rhubarb were measured using HPLC. The geographic distributions were collected using GPS and the nutrients in the soil were measured using the methods in the literature. The results showed that the levels of major API vary significantly among plants of different locations according to variance analysis. The species factor has few obvious effect on the overall properties of the rhubarb by the cluster analysis because of the two source species occurring in all divided three groups. However, *Rheum tanguticum* Maxim.ex Balf. is less effective at synthesizing and accumulating 9 API out of 14 than *Rheum palmatum* L. The correlation analysis and regression analysis also indicated that a lower latitude should be considered in the accumulation of API and a lower longitude should be considered to produce more compound anthraquinones. Lower levels of total P, rapidly available P and available molybdenum (Mo) and higher available K and available Zn in the soil were significantly correlated to accumulation of API in rhubarb. These results provide a basis for the clinical application and controlling the levels of the major API of rhubarb during artificial cultivation.

## Introduction

Rhubarb is the radix and rhizome of *Rheum officinale* Baill., *R*. *palmatum* L. and *R*. *tanguticum* Maxim.ex Balf. and is a commonly used raw material for crude drugs in China, Japan, Europe, America and Southeast Asia [1]. Modern pharmacology has demonstrated that rhubarb can cure constipation, hyperlipidaemia, hepatic injury and diabetes[2–5]. Chemical analysis shows that rhubarb contains anthraquinones, tannins, polyose, phenylbutazones, and stilbenes [6–9]. Relevant studies show that different active pharmaceutical ingredients (API) have different pharmacological activities, for example, emodin has effects in preventing viral infections, tumor, atherosclerosis, fungal infections and allergies [10–12]. Rhein can inhibit blood vessel hyperplasia [13]. Sennoside A functions in lavation [14] and emodin-8-0-D-glucopyranoside functions in promoting intelligence [15].

**Fig 1:**
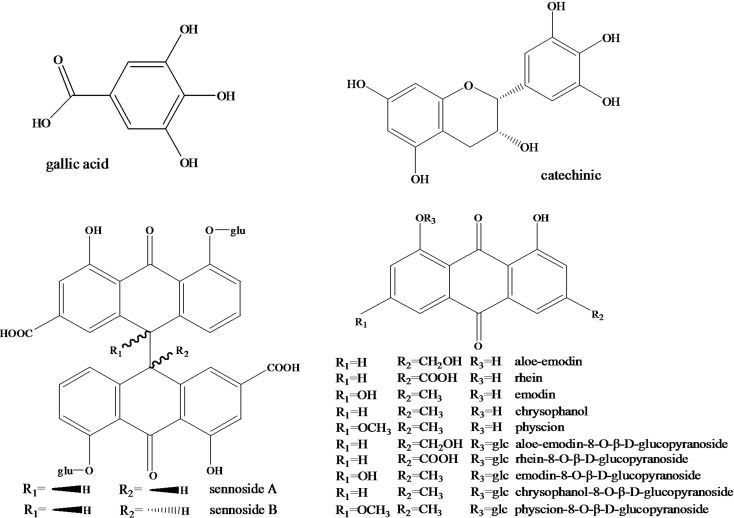
Chemical structures of the 14 active pharmaceutical ingredients (API) of rhubarb.

In China, *R*. *palmatum*, *R*. *tanguticum* and *R*. *officinale* are the official source species of rhubarb [16]. All the species are mainly distributed in the southwest of China [17]. Previous reports addressed the contents of major API of rhubarb from different locations discovering that the compound anthraquinones varied among different locations [18]. Compound anthraquinones includes aloe-emodin-8-O-β-D-glucopyranoside (AE8G), rhein-8-O-β-D-glucopyranoside (R8G), emodin-8-O-β-D-glucopyranoside (E8G), chrysophanol-8-O-β-D-glucopyranoside (C8G), physcion-8-O-β-D-glucopyranoside (P8G). In addition to the above-mentioned ingredients, the major API of rhubarb also include the polyphenols gallic acid (GA) and catechinic (CA); the dianthrone glycosides sennoside A (SA) and sennoside B (SB) and the free anthraquinones aloe-emodin (AE), rhein (RH), emodin (EM), chrysophanol (CH) and physcion (PH). The chemical structures of the 14 API were listed in Fig 1. Therefore, the previous reports only focused on differences in a subtype of ingredients and lacked an overall evaluation of API of rhubarb. In addition, the macro-elements, secondary elements and micro-elements in the soil are very important for forming the structural and functional ingredients that are vital for plants. However, few reports about the influence of soil on the levels of major API in rhubarb are available.

To perform an overall evaluation of the contents of major API in rhubarb and analyze influencing factors such as geographic distribution, soil and species, 128 samples of wild materials including *R*. *palmatum* and *R*. *Tanguticum* were collected from 25 locations in 5 major rhubarb producing areas in China, together with soil samples, geographic locations and species information. The wild resource of *R*. *officinale* was not collected in this work because of rareness. HPLC was employed to detect the contents of fourteen API in rhubarb. Then, variance analysis and cluster analysis were applied to evaluate the correlation between the levels of major API and influencing factors such as geographic distribution, soil and species. To ensure the identification of the factors exerting the greatest influence, the correlation analysis and regression analysis were applied while minimizing multicollinearity and spatial autocorrelation. This work will provide a reference for controlling the levels of major API in rhubarb during cultivation.

## Results

### Analysis of the contents of fourteen API in rhubarb from different locations

**Fig 2:**
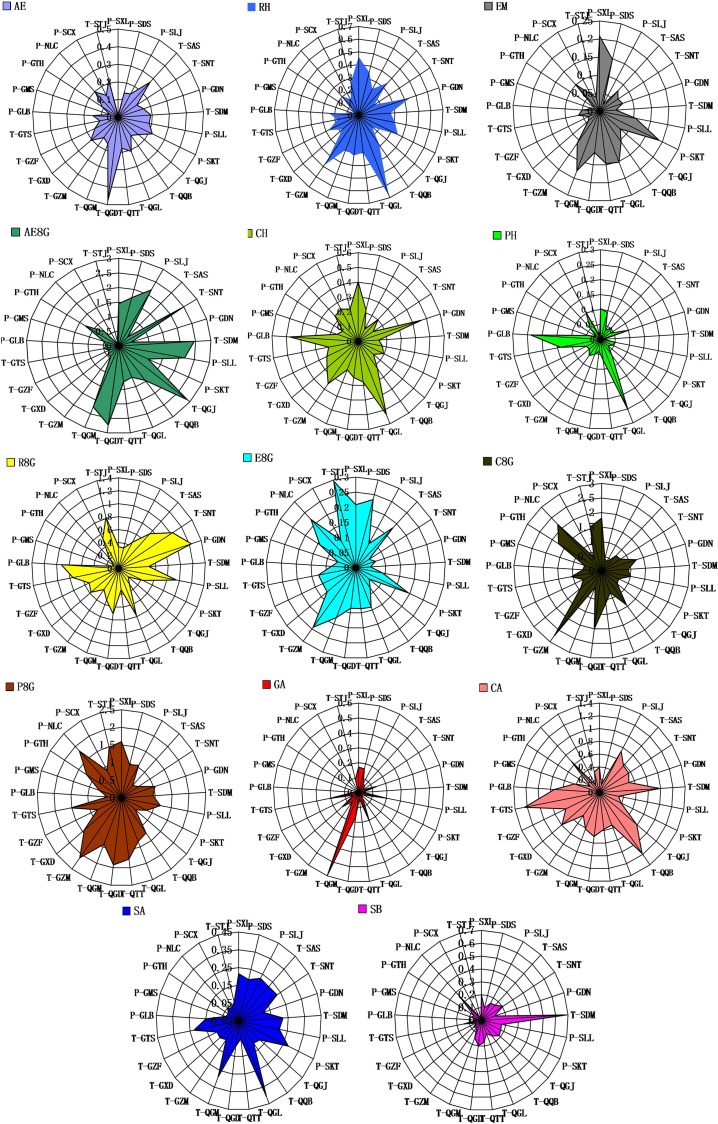
Comparison of the contents of API in rhubarb from different locations.

According to variance analysis, the content of each ingredient varied extremely significantly among different locations. GA presented the greatest difference, with the highest content (0.5941%) and the lowest content (0.0096%); the highest content was 61.89 times of the lowest. P8G showed the least difference, with the highest content (2.11%) and the lowest content (0.42%); the highest content was 5.02 times of the lowest. The ratios of the highest content to the lowest content for the other twelve API, AE, RH, EM, CH, PH, AE8G, R8G, E8G, C8G, CA, SA and SB were 58.92 times, 13.53 times, 11.09 times, 5.61 times, 24.88 times, 46.81 times, 13.43 times, 6.02 times, 7.57 times, 9.72 times, 16.96 times, and 43.70 times, respectively (Fig 2). This indicates that contents of major API in rhubarb varied from different locations.

### Cluster analysis of rhubarb from different locations based on 14 API

**Fig 3:**
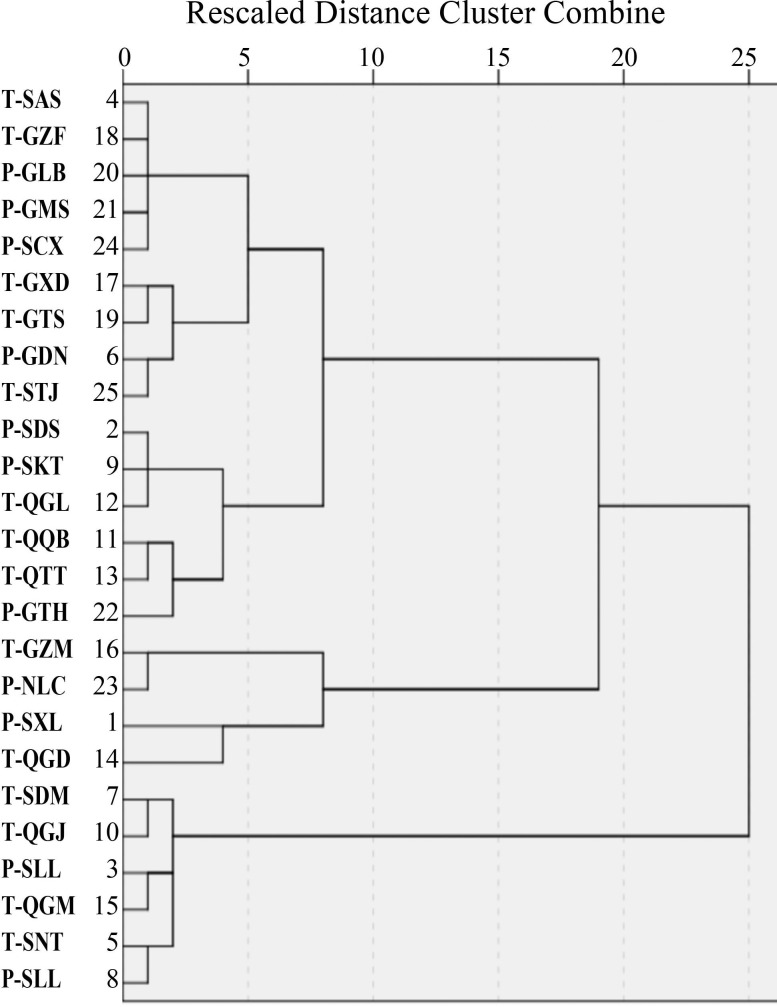
Cluster analysis of the contents of API in rhubarb from 25 locations.

In cluster analysis, the 25 locations were divided into three groups. The measured levels of major API of 128 materials from 25 locations show that *R*. *tanguticum* and *R*. *palmatum* cannot be divided by their overall composition. In addition, *R*. *Tanguticum* and *R*. *palmatum* occur in all three groups, indicating that the species factor has little influence on the API of rhubarb (Table 1 and Fig 3).

**Table 1:** Regression analysis results for the influencing factors and 14 API in rhubarb.

	AE	RH	EM	CH	PH	AE8G	R8G	E8G	C8G	P8G	GA	CA	SA	SB
*R*^2^	0.239	0.514	0.512	0.577	0.552	0.688	0.32	0.195	0.356	0.423	0.379	0.284	0.626	0.568
Longitude	-1.25	12.15	0.74	12.01	6.09	30.58**	-52.81**	-22.04*	-30.94**	-27.24**	21.92	7.28	4.54	-6.5
Latitude	-14.59*	-15.72**	-10.48*	-6.69	-10.71**	-2.24	-39.70**	-6.1	-5.48	-9.87**	-17.03*	-2.12	-18.44**	-0.66
Altitude	-1.12	0.46	0.56	1.05*	-1.2	-0.56	0.01	0.68	-0.39	-0.31	0.85	0.38	0.15	-0.93
Total N	-0.3	-0.53**	-0.04	-0.52**	-0.84**	0.88**	-1.35**	0.1	-0.05	-0.02	-0.93**	-0.06	-0.46**	0.57**
Total P	-1.98*	-2.58**	-1.86**	-2.16**	-4.54**	1.59*	-2.09*	-0.61	0.71	0.52	-2.09*	-0.01	-2.03**	1.49
Total K	0.7	0.25	-0.7	0.08	3.68*	1.05	-0.23	-0.95	-0.97	-1.91**	0.25	0.91	2.38**	0.84
Available N	0.48*	0.3	-0.27	0.36	0.8**	-0.85**	0.41	-0.2	-0.73**	-0.71**	1.10**	-0.37	0.48*	-0.70*
Available P	0.21	-0.11	0.1	-0.14	0.76**	0.1	-0.45*	0.22	-0.38**	-0.35**	0.25	-0.2	0.40**	-0.03
Available K	0.11	0.75*	0.04	0.69*	1.37*	-0.94*	1.59**	-0.4	0.22	0.54	0.25	0.08	0.23	-0.29
PH	-1.92*	0.16	-0.54	-0.61	-4.69**	-1.13	1.08	-0.36	-0.02	0.59	-1.34	0.07	-1.76**	-1.5
Available Mg	-0.71	-0.07	-0.85**	0.17	0.94*	-0.41	0.81*	-0.49	-0.92**	-0.82**	-0.38	1.11**	0.60*	-0.86**
Available Cu	-0.12	-0.44**	-0.25	-0.40**	-0.1	0.85**	-0.34	-0.36	-0.67**	-0.62**	0.24	-0.11	0.81**	0.13
Available Mn	-0.48	-0.42	0.04	-0.65**	-1.07*	0.70*	-1.19**	-0.03	0.17	-0.17	-0.87	0.01	-0.35	0.32
Available Zn	0.36	0.84**	0.37*	0.68**	0.84**	-0.71**	1.21**	0.33	0.39*	0.40**	0.98**	0.04	0.35*	-0.35
Available B	-0.07	-0.05	0.19*	0.06	-0.31	0.08	-0.45**	0.21*	0.35**	0.31**	0.03	-0.05	-0.32**	0.05
Available Mo	-0.74	-0.5	-0.58*	-0.16	-0.9	0.46	-1.32**	-0.34	-0.62*	-0.93**	-0.74	-0.12	-0.66*	-0.70*
Species	-0.37*	-0.36**	-0.35**	0.01	-0.1	0.05	-0.51**	-0.42**	-0.35**	-0.30**	-0.42*	-0.09	-0.34**	0.21

Note: Spatial mixed linear models were performed on API of rhubarb against the key impact factors in the ridge regression models.

* *P* < 0.05

** *P* < 0.01, *R*^2^ = adjusted coefficient of determination.

### Influence of geographic distribution, soil and species on API in rhubarb

As shown in Table 1, the latitude exerted negative influence on AE, RH, EM, R8G, P8G, GA and SA. While longitude only exerted significant influence on 5 compound anthraquinones with negative influence on R8G, E8G, C8G, P8G and positive influence on AE8G. Altitude only had positive influence on the accumulation of CH.

Species exerted significant negative influence on AE, RH, EM, R8G, E8G, C8G, P8G, GA and SA, indicating that *R*. *tanguticum* is less effective at synthesizing and accumulating of these ingredients than *R*. *palmatum* (Table 1).

As for influence of soil, total N had negative influence on RH, CH, PH, R8G, GA, SA and positive influence on AE8G and SB, while available N only had negative influence on AE8G, C8G, P8G and positive influence on GA and SA. Total P had negative influence on AE, RH, EM, CH, PH, R8G, GA and SA and positive influence on AE8G, while available P had negative influence on R8G, C8G, P8G and positive influence on PH and SA. Total K exerted negative influence on P8G and positive influence on PH and SA, while available K exerted negative influence on AE8G and positive influence on RH, CH, PH and R8G.PH value negatively affected AE, PH and SA. Available Mg negatively affected EM, C8G, P8G and SB and positively affected PH, R8G, CA and SA. Available Cu negatively affected RH, CH, C8G and P8G and positively affected AE8G and SA. Available Mn negatively affected CH, PH and R8G and positively affected AE8G. Available Zn negatively affected AE8G and positively affected RH, EM, CH, PH, R8G, C8G, P8G, GA and SA. Available boron (B) negatively affected R8G and SA and positively affected EM, C8G, E8G and P8G. Available molybdenum (Mo) negatively affected EM, R8G, C8G, P8G, SA and SB.

## Discussion

In plants, the production and accumulation of secondary metabolites are major mechanisms of adaptation to environmental stresses [19]. Rhubarb, a type of medicinal plant, may have developed its capability to produce and accumulate secondary metabolites such as free anthraquinones, phenolic acids and bianthrone glycosides to adapt to its environment. Accordingly, the production and accumulation of secondary metabolites in rhubarb are greatly influenced by environmental and species factors.

### Influence of geographic distribution on accumulation of API in rhubarb

Geographic distribution shows the clearest influence on the contents of API. Among the five compound anthraquinones assessed in this work, longitude has significantly negative effects on four of them and positive effects on AE8G. Latitude exerts negatively effects on the levels of all major API in rhubarb. In China, rainfall decreases with decreasing longitude [20]. Increases in the content of soluble sugar and the osmotic pressure in cells are major mechanisms of drought resistance of plants [21–22]. Moreover, soluble sugar is indispensable for the production of compound anthraquinones [23]. Hence, influence of longitude on API in rhubarb, especially on the contents of compound anthraquinones, was indirectly produced through changes in rainfall and dryness.

### Influence of species on accumulation of API in rhubarb

The synthesis and accumulation of secondary metabolites are influenced by both environmental and species factors. In this work, cluster analysis on the measured levels of major API of 128 materials from 25 locations shows that *R*. *tanguticum* and *R*. *palmatum* cannot be divided by their overall composition. In addition, *R*. *tanguticum* and *R*. *palmatum* occur in all three groups defined in the cluster analysis, indicating that the two species have similar levels of the major API and that is the main reason for the two species to be chosen as the official original plant species[1]; the other species is *R*. *officinale*, but the wild resources for this species are rare. Regression analysis shows that species had significant negative correlations with the 9 API out of 14, indicating that *R*. *tanguticum* is less effective at synthesizing and accumulating these ingredients than *R*. *palmatum*.

### Influence of soil components on accumulation of API in rhubarb

Among the fifteen soil components and qualitative factors in the work, organic matter and available iron were excluded from the regression analysis to avoid spatial multicollinearity and autocorrelation, respectively. Except organic matter and available iron, other involved factors showed clearly influence on at least one API in rhubarb.

In soil, total N includes insoluble organic N, soluble organic N and inorganic N (hydrolysable N), which can all be converted to one another through activities of the root system and soil microbes [24]. Thus, the influence on the contents of major API in rhubarb from total N cannot be studied without combining both the total N and hydrolysable N. This work shows that the influence of total N on rhubarb API is markedly different from that of hydrolysable N: they have different or even opposite effects on all API. The results in this work demonstrate that influence on accumulation of API from total N primarily attributes to the fact that rhubarb is a high-altitude plant that lives under a relatively low temperature for most of its growing season. Under low temperatures the plant will preferentially absorb soluble organic N [25], which is influenced by the transporter and is related to energy [26]. When rhubarb mainly absorbs organic N, the activity of the transporter and consumption of energy may become a barrier for the synthesis and accumulation of other API in rhubarb, thereby reducing the accumulation of other secondary metabolites in rhubarb. Therefore, the influence on the contents of major API in rhubarb from total N may be primarily exerted through active absorption of soluble organic N by rhubarb. In present work, it can be concluded that Total N and Available N affect the accumulation of some API.

This work also showed that the effect of total P on the contents of major API in rhubarb differs from that of rapidly available P. P is a component of nucleic acids in plants and plays a vital role in energy transfer and breeding activities. In soil, however, P can be easily absorbed to the soil particle surface or can form phosphates with some substances (Ca, Mg, Fe, Al, Mn, Cu, etc.) in soil to generate the immobile P [27]. Insoluble P and rapidly available P are collectively called total P, which represents the size of the P pool in the soil. The available P represents the level of P that can be absorbed by crops. The difference between the influence of rapidly available P and that of total P on the levels of major API in rhubarb is primarily caused by the immobile and insoluble P. Except positive correlation of PH and SA with rapidly available P and AE8G with total P, less rapidly available P and total P are more beneficial to the accumulation of API.

K, as one of the three macro-elements, can be divided into the slowly available K and rapidly available K in soil. The influence of rapidly available K may influence the contents of major API in rhubarb such that when the content of rapidly available K in soil increases, the K passively absorbed by rhubarb increases, thereby raising the K content in rhubarb, facilitating the transportation of substances in rhubarb, affecting enzyme activities [28] and enabling the synthesis and accumulation of rhubarb API, ultimately accelerating the accumulation of many active ingredients. In present work, except AE8G, more rapidly K are more beneficial to the accumulation of API.

Zn is an important microelement for plants; it is indispensable for forming nucleic acid and proteins and forms part of the structures of multiple enzymes. It also has catalytic action towards enzymes [29], indicating that Zn may promote the synthesis and accumulation of API by influencing the structure or catalytic efficiency of certain enzymes during the metabolic process. In present study, except AE8G, more available Zn are more beneficial to the accumulation of API. Available Mg and available B also affect the accumulation of some API. Available Mo has negative influence on the synthesis and accumulation of API. Hence, in terms of choosing the cultivation base for rhubarb, the soil should have a lower Mo concentration.

## Conclusion

For the first time, we conducted a study to analyze the relationship between the levels of API in rhubarb and various factors including species, geographic distribution and soil. The environmental and species factors demonstrated substantial influence on the levels of API in rhubarb. Species showed no obvious influence on major API but *R*. *palmatum* performs better in producing and accumulating API than *R*. *tanguticum*. Hence, *R*. *palmatum* could be used as a source material if a higher content of API is required. Regarding the influence of geographic distribution, it is concluded that a lower latitude should be considered in the accumulation of API and a lower longitude should be considered to produce more compound anthraquinones. The correlation analysis and regression analysis also showed that lower levels of total P, rapidly available P and available molybdenum (Mo) and higher available K and available Zn in the soil were significantly correlated to the accumulation of API in rhubarb. In further work, we will consider more ecological factors on the accumulation of API in rhubarb.

## Materials and Methods

### Ethics Statement

No specific permission was required for the experimental locations or activities because the land accessed was not privately owned or protected and the experimental activities had no negative impacts on the land and the environment. No approval was required from the Plant Protection Committee because the work did not involve endangered or protected species.

### Plant materials

The 128 samples used in this work were collected in 2013 from natural growing plots from 25 counties in 5 provinces. With the prerequisite of protecting the local germplasm resources and ecological environment and to ensure that samples were representative, 5–7 plants were collected from each location, with a distance of more than 50 m between any two plants. *R*. *officinale* was not collected in present work because of rareness. The location, herbarium, altitude, latitude and longitude information for all materials are shown in Table 2. The species of the plant were identified by Shengli Wei.

**Table 2:** Locations, species, altitude, latitude and longitude of materials.

Sample	Location	Species	Longitude	Latitude	Altitude (m)	Cluster analysis (Group)
P-SXL	Xiaojin,Sichuan	*R*. *palmatum*	E102°26′50.81″	N31°28′07.06″	4312	Ⅱ
P-SDS	Danba,Sichuan	*R*. *palmatum*	E101°32′55.86″	N30°51′35.83″	3636	Ⅰ
P-SLJ	Litang, Sichuan	*R*. *palmatum*	E100°15′09.17″	N30°13′35.91″	4265	Ⅲ
T-SAS	Songpan, Sichuan	*R*. *tanguticum*	E103°34′54.37″	N32°49′32.84″	3282	Ⅰ
T-SNT	Ruoergai, Sichuan	*R*. *tanguticum*	E102°27′41.74″	N33°21′11.73″	3447	Ⅲ
P-GDN	Diebu, Gansu	*R*. *palmatum*	E103°39′40.62″	N34°05′41.26″	3227	Ⅰ
T-SDM	Dege, Sichuan	*R*. *tanguticum*	E099°10′28.20″	N31°56′43.83″	3934	Ⅲ
P-SLL	Luhuo, Sichuan	*R*. *palmatum*	E100°39′56.91″	N31°23′54.51″	3166	Ⅲ
P-SKT	Kangding, Sichuan	*R*. *palmatum*	E101°45′40.80″	N30°13′36.41″	4077	Ⅰ
T-QGJ	Jiuzhi, Qinghai	*R*. *tanguticum*	E101°30′29.05″	N33°30′33.29″	3649	Ⅲ
T-QQB	Qilian, Qinghai	*R*. *tanguticum*	E100°12′59.18″	N38°09′36.25″	2981	Ⅰ
T-QGL	Guide, Qinghai	*R*. *tanguticum*	E101°14′37.38″	N36°14′04.01″	3728	Ⅰ
T-QTT	Tongde, Qinghai	*R*. *tanguticum*	E100°27′08.02″	N35°04′23.22″	3728	Ⅰ
T-QGD	Dari, Qinghai	*R*. *tanguticum*	E099°42′15.39″	N33°44′38.56″	3981	Ⅱ
T-QGM	Maqin, Qinghai	*R*. *tanguticum*	E100°14′53.82″	N34°27′50.02″	3746	Ⅲ
T-GZM	Zhuoni, Gansu	*R*. *tanguticum*	E103°30′47.44″	N34°34′33.39″	3558	Ⅱ
T-GXD	Xiahe, Gansu	*R*. *tanguticum*	E102°38′35.08″	N35°10′06.40″	3360	Ⅰ
T-GZF	Zhouqu, Gansu	*R*. *tanguticum*	E103°28'59.11"	N34°31'59.10"	3000	Ⅰ
T-GTS	Tianzhu, Gansu	*R*. *tanguticum*	E102°53′17.46″	N36°58′30.33″	3098	Ⅰ
P-GLB	Lixian, Gansu	*R*. *palmatum*	E104°52′52.12″	N33°57′16.91″	2136	Ⅰ
P-GMS	Minxian, Gansu	*R*. *palmatum*	E104°06′54.69″	N34°23′01.86″	2530	Ⅰ
P-GTH	Tanchang, Gansu	*R*. *palmatum*	E104°43′50.33″	N33°57′17.50″	2331	Ⅰ
P-NLC	Longde, Ningxia	*R*. *palmatum*	E106°07′01.69″	N35°33′28.71″	2358	Ⅱ
P-SCX	Longxian, Shaanxi	*R*. *palmatum*	E106°38′45.02″	N35°51′14.43″	1441	Ⅰ
T-STJ	Taibai, Shaanxi	*R*. *tanguticum*	E107°58′38.12″	N33°59′38.12″	2833	Ⅰ

### HPLC assessment of major API in rhubarb

Analyses were performed on an Agilent 1200 Series liquid chromatograph (Agilent Technologies; Palo Alto, CA, U.S.A), consisting of G1311A Quat Pump and a G1329A HiP-ALS SL auto-injector connected to a G1315B UV detector. Samples were separated on an AGILENT ZORBAX SB-C_18_ column (5 μm, 4.6×250 mm). The reference material for GA, CA, AE, RH, EM and CH were obtained from Chengdu Biopurify Phytochemicals Ltd. (Chengdu, China). The SA, SB and PH reference material were obtained from National Institutes for Food and Drug Control of China. All reference material had a purity of at least 98%.

The extraction and quantitative analysis of polyphenols, dianthrone glycosides, free anthraquinones: Dried samples (1 g) were extracted with 60% methanol (50 mL) for 60 min under ultrasonication (200 W, 40 MHz, 30°C), after which the solution was cooled to room temperature, adjusted with 60% methanol to the original volume, mixed and passed through a membrane filter (pore size, 0.22 *μ*m). A volume of 10 *μ*L was used for HPLC analysis. The mobile phase consisted of HPLC-grade water containing 0.05% H_3_PO_4_(A) and acetonitrile (B). The following gradient profile was used: 0 min, 5% B; 10 min, 11% B; 30 min, 15% B; 45 min, 17% B; 60 min, 22% B; 75 min, 36% B; 90 min, 60% B; 110 min, 60% B; and 125 min, 5% B. The API were quantified based on the peak areas at 254 nm in the UV spectrum. The flow rate was 1.0 mL/min, and the column temperature was 40°C.

**Fig 4:**
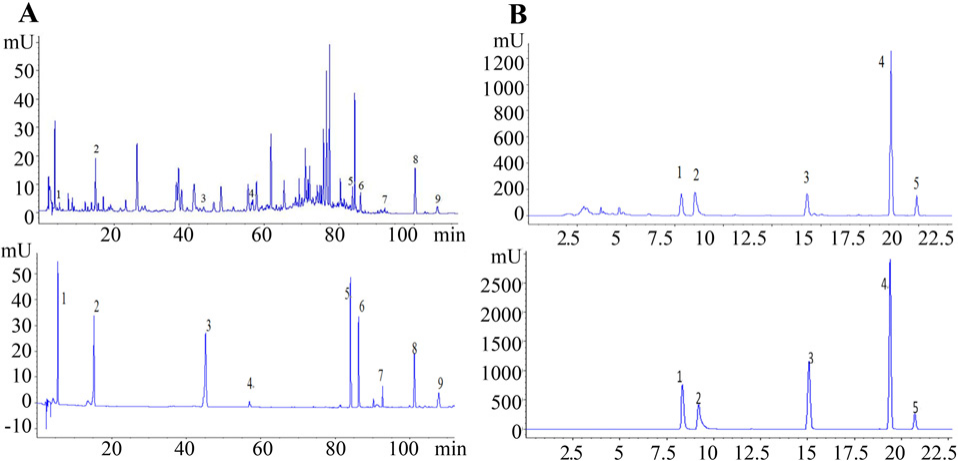
A: Chromatograms of rhubarb and the corresponding reference material of GA (1), CA (2), SB (3), SA (4), AE (5), RH (6), EM (7), CH (8), and PH (9). B: Chromatograms of rhubarb and the corresponding reference material of AE (1), RH (2), EM (3), CH (4), and PH (5).

Total anthraquinones were extracted and analysed according to the methods in the Pharmacopoeia) [16]: A volume of 20 *μ*L was used for HPLC analysis. The mobile phase consisted of HPLC-grade water containing 0.05% H_3_PO_4_ (A) and acetonitrile (B). The following gradient profile was used: 0 min, 50% B; 6 min, 53% B; 10 min, 58% B; 14 min, 70% B; 16 min, 85% B; 20 min, 85% B and 22 min, 50% B, 50% B. The API were quantified based on the peak areas at 254 nm in the UV spectrum. The flow rate was 1.0 mL/min, and the column temperature was 20°C. Chromatograms of rhubarb and corresponding reference materials were shown in Fig 4.

The contents of various API were obtained according to standard curve of reference material prepared as follows:

### Free anthraquinones, polyphenols and dianthrone glycosides

The stock solutions of the free anthraquinones were prepared by dissolving accurately-weighed portions of the standards in 60% methanol. The concentrations of the compounds were as follows: 0.1216 mg/mL (GA), 1.04 mg/mL (CA), 0.8 mg/mL (SA), 0.503 mg/mL (SB), 0.234 mg/mL (AE), 0.246 mg/mL (RH), 0.042 mg/mL (EM), 0.165 mg/mL (CH) and 0.129 mg/mL (PH). The standard solution was further diluted into a series of different concentrations. Ten *μ*L of each concentration was analyzed to form the regression equation (S1 Table).

### Anthraquinones

The stock solutions of the anthraquinones were prepared by dissolving accurately-weighed portions of the standards in methanol. The concentrations of the compounds were as follows: 0.435 mg/mL (AE8G), 0.852 mg/mL (R8G), 0.596 mg/mL (E8G), 0.888 mg/mL (C8G) and 0.3756 mg/mL (P8G). The standard solution was further diluted into a series of different concentrations. Twenty *μ*L of each concentration was analyzed to form the regression equation shown (S2 Table).

### Collection of soil samples and measuring method of main nutrient elements

One soil sample was taken from the place where a plant sample was collected. During sampling, 0–40 cm soil was collected and evenly blended. Then, 2 kg soil was put into the sample sack with the quartering method and brought to the lab. Soil chemical properties, including pH, organic matter, total N, total P, total K, available N, available P, available K, available Mg, available iron (Fe), available Mn, available Cu, available Zn, available Mo and available B, were determined according to standard procedures described in the standard handbook of soil testing [30].

### Collection of main geographic information

Geographic information consists of altitude, longitude and latitude, which were recorded using GPS (heacnet, HC 608) during sampling.

### Data analysis

All data were processed with Excel, and variation analysis, cluster analysis, correlation analysis and regression analysis were performed using SPSS (19.0). For the convenience of quantitative analysis, pseudovariables were introduced for the species *R*. *tanguticum* (1) and *R*. *Palmatum* (0). To avoid significant differences in the order of magnitude between variables (API, geographic distribution, soil, species), all values were log-transformed for the correlation analysis and regression analysis. In the correlation analysis, to eliminate the influence of multicollinearity during the regression analysis, independent variables with individual correlation values greater than 0.8 against other independent variables were abandoned. The preliminary regression analysis shows that the variance inflation factor (VIF) of organic matter is greater than 100. To eliminate the influence of spatial autocorrelation and ensure statistical meaning of correlation analysis and regression analysis, organic matter and available iron were excluded from the regression analysis. We aimed to address the factors with the most influence in the statistical analysis and to minimize the Durbin-Watson (DW) value. To that end, the backward regression analysis was used in building the regression equation. *P* < 0.05 was recognized as significance.

## Supporting Information

S1 Table Calibration curves of the nine biological active ingredients. (DOCX)

S2 Table Calibration curves of the five biological active ingredients. (DOCX)
