# Supplementary material for: Influence of the Environmental Factors on the Accumulation of the Bioactive Ingredients in Chinese Rhubarb Products
Source: PLoS One. 2016 May 3;11(5):e0154649. doi: 10.1371/journal.pone.0154649 (PMC4854418; doi:10.1371/journal.pone.0154649)
Supplement: S1 Table — (DOCX) [file pone.0154649.s001.docx]

Table S1. Calibration curves of the nine biological active ingredients

| Active compounds | Regression equations | The linear range (*μ*g) | Correlation coefficient (*R*^2^) |
| --- | --- | --- | --- |
| Gallic acid | y = 1599.70x-4.47 | 0.01-1.22 | 1.00 |
| Catechinic | y = 150.99x-2.79 | 0.04-10.40 | 1.00 |
| Sennoside B | y = 381.07x+2.79 | 0.02-5.03 | 1.00 |
| Sennoside A | y = 345.68 x +11.46 | 0.03-8.00 | 1.00 |
| Aloe-emodin | y =3875.50 x+27.89 | 0.01-2.34 | 1.00 |
| Rhein | y = 2145.50x+41.17 | 0.01-2.46 | 1.00 |
| Emodin | y =3131.30x+2.91 | 0.00-0.42 | 1.00 |
| Chrysophanol | y =4303.10 x+22.70 | 0.00-1.65 | 1.00 |
| Physcion | y = 1642.50x +9.87 | 0.00-1.29 | 1.00 |
