# Supplementary material for: Influence of the Environmental Factors on the Accumulation of the Bioactive Ingredients in Chinese Rhubarb Products
Source: PLoS One. 2016 May 3;11(5):e0154649. doi: 10.1371/journal.pone.0154649 (PMC4854418; doi:10.1371/journal.pone.0154649)
Supplement: S2 Table — (DOCX) [file pone.0154649.s002.docx]

Table S2. Calibration curves of the five biological active ingredients

| Active compounds | Regression equations | The linear range (*μ*g) | Correlation coefficient (*R*^2^) |
| --- | --- | --- | --- |
| Aloe-emodin | y =3621.00 x+48.07 | 0.02-1.74 | 1.00 |
| Rhein | y = 1115.50x+17.03 | 0.03-4.26 | 1.00 |
| Emodin | y =3581.50x +56.76 | 0.03-2.98 | 1.00 |
| Chrysophanol | y =3640.80 x+108.75 | 0.03-4.44 | 1.00 |
| Physcion | y = 1775.00 x -14.75 | 0.02-1.88 | 1.00 |
